# Green HEMS in mountain and remote areas: reduction of carbon footprint through drones?

**DOI:** 10.1186/s13049-023-01099-5

**Published:** 2023-07-18

**Authors:** Michiel J. van Veelen, Giacomo Strapazzon

**Affiliations:** 1 Institute of Mountain Emergency Medicine, Eurac Research, Bolzano, Italy; 2 Department of Sport Science, Medical Section, University of Innsbruck, Innsbruck, Austria

To the editor,

We read with interest great interest the commentary by ter Avest et al. We applaud their proposition to promote a green Helicopter Emergency Medical Services (HEMS) approach [1]. The use of helicopters and HEMS missions is widespread in mountain and remote areas where mission times are longer than in urban areas [2] Therefore, some aspects could benefit from an additional adaption of the measures proposed. When we apply the “6R” attitude to these HEMS missions, most carbon emission reduction can be achieved by assessing the *rethink*, *refuse*, and *reduce* aspects.

Rethink: Using unmanned aerial vehicles (UAVs) or drones can play a substantial role in carbon reduction. A typical quadcopter drone currently used by mountain rescue services in the Alps carrying a 0.5 kg payload has an energy consumption of around 0.08 MJ/km, which corresponds to a carbon footprint of 12.1–23.5 g of CO_2_/km (depending on the modality of electricity generation) [3]. This yields a carbon footprint of 250–500 g of CO_2_ for a 40-min flight compared to 720 kg of CO_2_ for a typical 50-min HEMS flight [1]. If a drone fully substitutes a helicopter, it could lead to a potential 1500 to 3000-fold reduction in carbon footprint. A drone of this type typically has an endurance of around 40 min and an airspeed of 10 m/s.

Refuse: HEMS teams in search and rescue missions are frequently deployed for the assessment of the scene safety of ground-based personnel in unclear and difficult-to-reach areas. For example, 50% of the avalanche rescue operations in Norway did not involve any victims [4]. Drones could assist in the assessment of the scene, the patient search, and the delivery of medical equipment [5]. Effective drone deployment could lead to a HEMS stand-down in case of no victims or provide an ongoing aerial overview of complex multiple incident management [6].

Reduce: In the European Alps, there are many HEMS providers to assist patients in a timely manner. Due to the large availability of standby helicopters and time-consuming ground-based alternative transport, even non-critically injured patients are assisted, contributing to a large carbon footprint. Drones can facilitate remote triage by assessing vital signs or establishing telemedical communication with a patient or bystander [5, 7] This information could lead to a HEMS stand-down or down triage to a ground-based rescue. Objective triage criteria for HEMS-assisted operations should be enforced to limit overuse, and in select non-critically injured patients, we should accept longer transport times.
